# The Book World of Medicine and Science

**Published:** 1900-01-06

**Authors:** 


### The Book World of Medicine and Science.

Surgery: A Treatise for Students and Practitioners. By Pickering Pick. (London: Longmans, Green, and Co. 1899. Price 25s.)

There is no doubt that Mr. Pick has done well in publishing this book, and in giving to the surgical students of St. George's Hospital such a handsome memento of his work in the hospital and so carefully written a handbook of the subject of their studies. Like many other books in surgery, the book begins with a chapter on inflammation, and its author, like those of other manuals, at once encounters the difficulty of finding a definition of the process which will not conflict with its "cardinal symptoms." Omitting paraphrase, the definition which he gives runs as follows: "I should regard inflammation as a modification of the normal physiological processes in the various tissues of the body, resulting from the application of some irritant . . . the processes in many instances being followed by the formation of a new material of a less highly organised nature than the original tissue in which the inflammation has taken place." Which, as a description of the process, is good; but we venture to think that the words "in many instances" invalidates it as a definition. If we are to omit the consequences which only follow "in many instances," and confine our attention entirely to what is stated to occur in all cases, we do not see how Mr. Pick's definition improves upon or even materially varies from that proposed by Sir John Burdon Sanderson, namely, "inflammation is the succession of changes which occur in a living tissue when it is injured, provided that the injury is not of such a degree as at once to destroy its structure and vitality. The phenomena of inflammation are, however, very well described, but whether it is well to place such results as "suppuration—ulceration—mortification," in quite a separate section, under the head of general diseases, is perhaps open to question. Clinically, as the student learns his work, they no doubt are to him separate and independent diseases; but one of the uses of a text book, as it seems to us, should be to correct that personification of diseases which almost inevitably arises when they are studied separately, just as they come in the course of a hospital visit. Not that Mr. Pick is by any means alone in thus dealing with his subject matter.

In the section devoted to general injuries we find a complete description of the different kinds of wounds and their treatment, including, of course, the antiseptic measures to be employed for the prevention of putrefaction and infection in wounds. Mr. Pick is evidently a great believer in drainage, which, perhaps, naturally goes with a certain degree of lack of faith in the possibility of attaining asepsis without the application of antiseptics to the wound. He says: "I think, on the whole, drainage is not practised as frequently as it should be. In my own personal experience I have more than once regretted not using a tube; I cannot say that I ever remember regretting the use of one. It seems to me that it is a wise precaution to take; it can do no harm, and the worst that can be said of it is that it necessitates the dressing of the wound on the second day, when, if no drainage tube had been used, the case would probably not have required dressing. But I am not sure that even this is an unmixed evil." As we have said, drainage and the use of antiseptics to the wound naturally go together, and we may point out that fully as Mr. Pick appreciates the advantages of asepsis, he seems to have his doubts about its being attainable with that certainty which is one of the articles of faith among some of our younger surgeons. He thus expresses his views: "The aseptic treatment of wounds may be summed up in two words: perfect cleanliness. Everything which is brought into contact with the wound is, or ought to be, absolutely pure and clean—that is to say, absolutely free from micro-organisms. . . . If this could be done perfectly, the system of aseptic surgery could be carried out in its entirety. But it is an exceedingly difficult, if not impossible, thing to bring about, and therefore it seem to me that it is fortunate that we have certain reliable chemical substances which are known to destroy these micro-organisms, and that by their use we give an additional security to our patients. The main objection to the 'antiseptic' treatment of wounds in contradistinction to the 'aseptic' treatment, is that the chemicals used in the former method are said to destroy not only the microbes but also many of the tissue elements. And this is no doubt true within certain limits. As we have already stated, the application of a strong solution of carbolic acid to a wound irritates it, and so causes a very considerable amount of serum to exude, and thus produces a condition of unrest, which is not favourable to union. So that ideally the aseptic treatment is the best; the difficulty is in carrying it out. And after all the amount of unrest is slight, and means may be taken for making provision for the escape of the effused fluid from the wound." These extracts will, we hope, serve to explain the exact position which Mr. Pick takes up in regard to asepsis. The best part of the book is that which is also by far the largest part of it, namely, that which is devoted to injuries and diseases of the special organs. These are admirably described, and everything which it is necessary that the student should know is placed before him in such a manner as greatly to facilitate its comprehension. It is easy to recognise the method and the power of expression of one well skilled in the art of teaching throughout the pages of this work, which, from its completeness, the freedom with which illustrations are introduced, and the sound teaching which it conveys, is, we think, sure to become a favourite with both students and practitioners, and especially with those who either are or have been connected with St. George's Hospital.

Herbert Fry's Royal Guide to the London Charities. Edited by John Lane. Thirty-sixth annual edition. (London: Chatto and Windus.)

This is an address book rather than a guide, seeing that the figures it contains relate to no defined period, and therefore convey no accurate idea to its readers.

